# The predictive role of the TAPSE/sPAP ratio for cardiovascular events and mortality in systemic sclerosis with pulmonary hypertension

**DOI:** 10.3389/fcvm.2024.1430903

**Published:** 2024-10-14

**Authors:** Marco de Pinto, Francesca Coppi, Amelia Spinella, Gianluca Pagnoni, Vernizia Morgante, Pierluca Macripò, Matteo Boschini, Anna Francesca Guerra, Francesca Tampieri, Ottavio Secchi, Martina Orlandi, Gabriele Amati, Federica Lumetti, Gilda Sandri, Rosario Rossi, Giuseppe Boriani, Anna Vittoria Mattioli, Clodoveo Ferri, Dilia Giuggioli

**Affiliations:** ^1^ Scleroderma Unit, Rheumatology Unit, Azienda Ospedaliero-Universitaria Policlinico di Modena, University of Modena and Reggio Emilia, Modena, Italy; ^2^ Cardiology Unit, Azienda Ospedaliero-Universitaria Policlinico di Modena, Modena, Italy; ^3^ Department of Medical and Surgical Sciences for Children and Adults, University of Modena and Reggio Emilia, Modena, Italy; ^4^ Department of Engineering, University of Modena and Reggio Emilia, Modena, Italy; ^5^ Internal Medicine and Centre for Hemochromatosis, University Hospital of Modena, University of Modena and Reggio Emilia, Modena, Italy; ^6^ Cardiology Division, Department of Biomedical, Metabolic and Neural Sciences, University of Modena and Reggio Emilia, Policlinico di Modena, Modena, Italy; ^7^ Settore Scienze Tecniche e Mediche Applicate Presso Alma Mater Studiorum, Università di Bologna, Bologna, Italy

**Keywords:** systemic sclerosis, pulmonary hypertension, pulmonary arterial hypertension, TAPSE, sPAP, interstitial lung disease, survival

## Abstract

**Introduction:**

Reduced TAPSE/sPAP ratio has recently emerged as a predictive parameter risk factor for PH, however its role in SSc has been poorly investigated. The aim of the study was to investigate the prognostic value of the TAPSE/sPAP ratio for the prediction of mortality and cardiovascular events in patients with SSc complicated by PH. A comparison between SSc patients with PAH (SSc-PAH) and those with PH and significant ILD (SSc-PH) was also carried out.

**Materials and methods:**

A retrospective single-center study in which all patients having SSc—complicated by PH—referring to the Scleroderma-Unit of the AOU Policlinico of Modena, from October 2013 to October 2023 were evaluated. All SSc patients underwent recurrent clinical examination, routine blood chemistry analysis, functional, instrumental evaluation.

**Results:**

61 SSc patients (F/M 52/9) were enrolled. During the follow-up, 60.1% of patients experienced at least one cardiovascular event and 62% died. The main causes of death were PH (39.4%) and other heart-related events (39.4%). The TAPSE/sPAP ratio was significantly lower in deceased patients compared to survivors (mm/mmHg 0.3 ± 0.12SD vs. 0.48 ± 0.17SD, *p* < 0.001). Compared to the SSc-PAH subgroup, the SSc-PH patients had lower survival rates (55.3 ± 31.2 SD months vs. 25 ± 19 SD, *p* = 0,05). At the multivariate analysis, TAPSE/sPAP ratio <0.32 mm/mmHg, male gender, and the presence of significant ILD were identified as independent predictors of mortality and cardiovascular events.

**Conclusion:**

Our work confirmed the predictive role of the TAPSE/sPAP ratio for mortality and cardiovascular events in patients with SSc complicated by PH.

## Introduction

Systemic sclerosis (SSc) is a rare connective tissue disease (CTD) of unknown etiology, characterised by immune-system alterations, diffuse microangiopathy and fibrosis involving the skin and internal organs (1, 2).

Pulmonary hypertension (PH) is a major cause of morbidity and mortality in SSc (3, 4) and patients with SSc and pulmonary arterial hypertension (PAH) have shown a higher mortality rate than patients with PAH related to other causes (4, 5).

All forms of PH may be observed in SSc, however it is mainly associated with group 1 PAH and group 3 PH (in presence of significant interstitial lung disease ILD) and less commonly with group 2 PH (6, 7). However, due to the varied and often mixed etiology underlying PH in SSc, the precise classification of the type of PH can be challenging (8).

The prevalence of PAH in SSc has been estimated to be between 10% and 19% (2, 9, 10) and SSc-PAH represents around 15%–30% of PAH in most registries. PAH is one of the leading causes of death in SSc, with two-year survival rates ranging from 64 to 89% (7, 11, 12). Moreover, prognosis seems to be worse in SSc patients with PH associated with ILD (13).

The tricuspid annular plane systolic excursion/systolic pulmonary arterial pressure (TAPSE/sPAP) ratio is emerging as a novel, non-invasive, easy to determine and reliable index of right ventricle- pulmonary artery (RV-PA) coupling. Several studies have shown a strong correlation with outcome, functional class and other hemodinamic indexes of PH and coupling, namely pulmonary vascular resistance (PVR) and pulmonary artery compliance (14, 15). In the 2022 ESC/ERS guidelines for PH, the TAPSE/sPAP ratio <0.55 mm/mmHg has been included among the additional echocardiographic signs indicative of PH, while a ratio <0.32 mm/mmHg and 0.19 mm/mmHg are included in the three-strata model risk assessment of mortality at 1 year (16).

However, the role of TAPSE/PAPs in SSc with PH has been poorly investigated.

The aim of the present study was to investigate the prognostic value of the TAPSE/sPAP ratio in predicting mortality and cardiovascular events in patients affected by SSc complicated by PH.

Secondary outcomes were:

1. Assessing the predictive role of other clinical, serological, hemodynamic and instrumental parameters and PAH specific therapies for mortality and cardiovascular events;
2. Comparing SSc patients with PAH (group 1, SSc-PAH) and PH associated with significant ILD (group 3; SSc-PH).

## Materials and methods

### Study population

**Figure 1:**
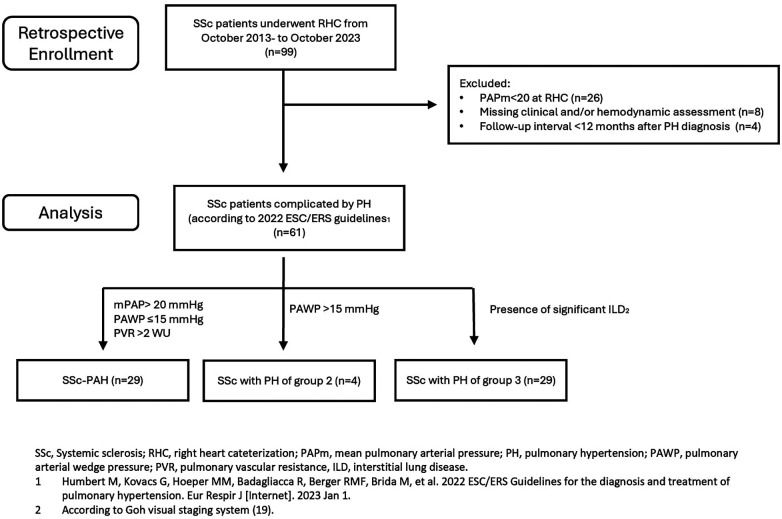
Enrollment population and analysis flow-chart.

In this retrospective single-center study we analysed all patients affected by SSc, both incident and prevalent, complicated by PH, and referring to the Scleroderma Unit of the AOU Policlinico of Modena, from October 2013 to October 2023. A flow-diagram of the retrospective enrolment of the study population is shown in Figure 1.

All patients satisfied the 2013 ACR/EULAR classification criteria for SSc (17). In all patients with suspected PH—according to the ESC/ERS guidelines (16, 18) and DETECT screening algorithm for PAH (6)—right heart catheterisation (RHC) was performed in order to confirm diagnosis. PH diagnosis and PH group classification were established according to the 2022 ESC/ERS guidelines (16). Patients who underwent RHC before 2022 were eventually diagnosed and reclassified following the aforementioned guidelines (16).

In particular, PAH was defined by the presence of precapillary PH in the absence of other causes (16); while PH of group 3 (16, 18, 19) was established on the basis of the presence of significant ILD, defined by fibrosis involving >20% of lung parenchyma and/or FVC <70% (according to Goh visual staging system).

Exclusion criteria were age <18 years, missing hemodynamic assessment and follow-up interval <12 months after PH diagnosis (confirmed at RHC).

The study was approved by the local ethics committee Area Vasta Emilia Nord (protocol no. 275/16). The study was performed in accordance with the Good Clinical Practice Guidelines and with the World Medical Association Declaration of Helsinki, revised in 2000, Edinburgh.

### Assessed parameters

All SSc patients underwent complete clinical, serological and instrumental evaluation within 12 months before RHC.

Disease duration was defined by the onset of the first non-Raynaud symptom. The presence of cardiovascular risk factors (smoking, diabetes, dyslipidemia, systemic hypertension, being overweight) and previous cardiovascular events (including heart failure, cardiac ischemic events, transient ischemic attack, stroke, arrhythmic events, sudden death) were assessed. Mortality, causes of death and new cardiovascular events were recorded. Survival time interval (months) was calculated from the date of RHC until death or the end of follow-up.

Pulmonary function tests (PFTs), including evaluation of forced vital capacity (FVC), total lung capacity (TLC) and 6 min walking test (6MWT) were performed. Presence or absence of desaturation (SpO2 <90%) and 6 min walking distance (6MWD) were also recorded. Diffusing capacity of the lung for carbon monoxide (DLCO) was obtained though the single breath method and then was adjusted for hemoglobin values and alveolar volume.

High resolution computed tomography (HRCT) of the thorax was performed for assessing the presence of ILD. ILD was further distinguished into limited or significant according to Goh visual staging system (19).

Echocardiographic measurements were performed by senior researchers using a commercially available system (Philips Epiq CVx). Ventricular diameters and wall thickness, left ventricular ejection fraction, TAPSE, and left and right atrium area were assessed by standard methods as recommended by the ASE/EACVI guidelines (20, 21). Systolic pulmonary arterial pressure (PAPs) was determined from the peak tricuspidal jet velocity (TRV) using the simplified Bernoulli equation and combined with an estimate of the RA pressure obtained by assessing the size and collapsibility of the inferior vena cava.

The TAPSE/sPAP ratio was then calculated for all patients and different thresholds (<0.55 mmHg, <0.32 mmHg and <0.19 mmHg) were further distinguished, according to the 2022 ESC/ERS guidelines (16). Taking into account the recent literature regarding TAPSE/sPAP in SSc (22), a cut-off of TAPSE/sPAP ratio <0.60 mmHg was further assessed. Subsequently we tested the different thresholds to evaluate their predictive value for mortality and cardiovascular events.

RHC was performed at the Cardiology Unit of the Policlinico of Modena, following standard methods (16). PAPm, pulmonary arterial wedge pressure (PAWP), pulmonary vascular resistance (PVR), cardiac output and cardiac index were recorded.

### Statistical analysis

All statistical analyses were performed using SPSS software (IBM). Continuous distributed variables were represented as median and standard deviation (SD) and categorical data were expressed as frequencies.

Categorical parameters were assessed using Chi-square test or Fisher’s exact test, while continuous variables were evaluated using Student’s *t*-test.

Univariate Cox regression analysis was applied to evaluate the significant variables for overall survival. Multivariate Cox regression analysis was performed on the significant variables in univariate Cox regression analysis. Hazard Ratio (HR) and 95% CI were reported.

Survival and cardiovascular events were represented by Kaplan-Meier curves and the log rank test was used for statistical comparisons.

Linear correlation was assessed by Pearson's coefficient. A significance level of 0.05 was used for all tests.

## Results

A total of 61 SSc patients (F/M-52/9, mean age at RHC of 66 years ± 10 SD) were enrolled. In particular, 7 patients with PAPm between 20 and 24 mmHg at RHC performed before 2022, were reclassified following the 2022 ESC/ERS guidelines.

Demographic and clinical characteristics are reported in Table 1.

**Table 1:** Demographic and clinical characteristics of SSc-PH patients.

Parameters	
Female (*n*,%)	85.2% (52/61)
Age (years)	66 ± 10
Smokers (*n*,%)	55.7% (27/61)
Diabetes	11,4% (7/61)
Systemic hypertension	39,3% (24/61)
Overweight (BMI ≥25)	6,5% (4/61)
Dyslipidemia	47,5% (29/61)
Previous cardiovascular events	29,5% (18/61)
Disease duration (years)	12.9 ± 8.4
Teleangiectasia	73.8% (45/61)
Digital ulcers (*n*,%)	55.7%% (34/61)
Cutaneous subset	
Limited	82% (50/61)
Diffuse	18% (11/61)
mRSS	8 ± 7
ILD (*n*,%)	77.0% (47/61)
Limited ILD	31.1% (19/61)
Significant ILD	45.9% (28/61)
NYHA stage	
I-II (*n*,%)	42.6,2% (26/61)
III-IV (*n*,%)	57.4% (35/61)
Autoimmunity	
ACA (*n*,%)	45.9% (28/61)
ATA (*n*,%)	17.9% (17/61)
ARA (*n*,%)	1.6% (1/61)
Electrocardiogram	
Right brunch block	45.9% (28/61)
Right axis deviation	11.5% (7/61)
Echocardiogram	
Left ventricle ejection fraction (%)	56 ± 5
Pericardial effusion (*n*,%)	14.8% (9/61)
Diastolic disfunction (*n*,%)	57.1% (16/61)
TRV (m/s)	3.3 ± 0.7
TAPSE (mm)	20 ± 3.5
sPAP (mmHg)	58 ± 22
TAPSE/sPAP ratio	0.39 ± 0.15
PFTs	
FVC (%)	84 ± 25
DLCO (%)	36 ± 16
FVC/DLCO ratio	2.5 ± 1,0
Desaturation at 6MWT	44.3% (27/61)
6MWD (mt)	391 ± 123
Videocapillaroscopy	
Aspecific (*n*,%)	0% (0/61)
Early (*n*,%)	11.5% (7/61)
Active (*n*,%)	37.7% (23/61)
Late (*n*,%)	50.8% (31/61)
Blood chemistry analysis	
BNP (pg/ml)	326 ± 347
Serum urate elevation (*n*,%)	44.3% (27/61)
RHC	*N* = 61
mPAP (mmHg)	35 ± 11
PVR (WU)	5.8 ± 3.6
PAWP	12.9 ± 6.4
PH group	
1 (*n*,%)	47.5% (29/61)
2 (*n*,%)	6.6% (4/61)
3 (*n*,%)	45.9% (28/61)
Outcomes	
Follow-up (months)	39.2 ± 29.0
Cardiovascular events (*n*,%)	60.1% (37/61)
Death (*n*,%)	62.3% (38/61)
Therapies	
Iloprost	73,8% (45/61)
CCbs (*n*,%)	52,5% (32/61)
Beta-blockers (*n*,%)	18% (11/61)
ERAs (*n*,%)	72,1% (44/61)
Bosentan (*n*,%)	45,9% (28/61)
Macitentan (*n*,%)	41% (25/61)
PDEi (*n*,%)	54,1% (33/61)
Selexipag (*n*,%)	6,6% (4/61)
Death causes	
Heart	19.6% (12/61)–39.4% (15/38)
PH	29.5% (15/61)–39.4% (15/38)
ILD	3.3% (2/61)–5.3% (2/38)
Infection	3.3% (2/61)–5.3% (2/38)
Cancer	6.6% (4/61)–10.6% (4/38)

PH, pulmonary hypertension; ILD, interstitial lung disease; mRSS, modified Rodnan Skin Score; NYHA, New York Heart Association; ANA, anti-nucleus antibodies; ACA, anti.centromere antibodies, ATA, anti-topoisomerase I antibodies; ARA, anti-RNA polymerase III antibodies; RV, right ventricle; TRV, tricuspid regurgitant velocity, TAPSE, tricuspid annular plane systolic excursion; sPAP, systolic pulmonary arterial pressure; TLC, total lung capacity; FVC, force vital capacity; DLCO, diffusing capacity of the lung for carbon monoxide; 6MWT, six minutes walking test, 6MWD, six minutes walking distance; BNP, brain natriuretic peptide; CCBs, calcium channel blockers; ERA, endothelin receptor antagonists; PDEi, phosphodiesterase inhibitors; mPAP, mean pulmonary arterial pressure; PVR, pulmonary vascular resistance; PAWP, pulmonary arterial wedge pressure; CpcPH, combined post- and pre-capillary PH.

Pre-capillary PH was detected in 81.9% (50/61) SSc patients, isolated post-capillary PH in 3.3% (2/61), and combined post- and pre-capillary PH in 14.8% (9/61).

According to the 2022 ESC/ERS guidelines, 47.5% (29/61) were classified as PAH (group 1), 6.6% (4/61) as group 2 PH, and 45.9% (28/61) as group 3 PH, due to the presence of significant ILD (16, 19). Mean SSc disease duration at the time of diagnosis of PH was 12.9 ± 8.4 years and in 80% of the patients (49/61) PH was diagnosed after the fifth year of disease. In one case, PH was the onset symptom of SSc. Most patients were treated with CCbs (52.5% or 32/61) and intravenous prostanoids (Iloprost, 73.8%, 45/61) for Raynaud phenomenon and digital ulcers, while 72.1% (44/61), 54.1% (33/61) and 6.6% (4/61) received therapy with ERAs (of which 45.9% with Bosentan and 41% with Macitentan), PDEi and/or Selexipag during the follow-up for treating PH, respectively.

During follow-up (39.2 months ± 29.0 SD, range 3–118 months), 37/61 (60.1%) SSc-PH patients experienced at least one (1.5 ± 1.6 SD) cardiovascular event: 57% (35/61) had heart failure, 16% (10/61) developed arrhythmias which required admission to hospital (7 atrial fibrillation, 1 complete atrioventricular block, 1 ventricular tachycardia), 5% (3/61) had a stroke/transient ischemic attack, 5% (3/61) had pulmonary thromboembolism and 11.5% patients (7/61) suffered sudden death. No cases of myocardial infarction were recorded.

Notably, among the whole population, 56% (35/62) of patients had at least one cardiovascular risk factor and 29.5% (18/61) had previous cardiovascular events (prior to PH diagnosis). However, as expected, only the 4 patients with PH of group 2 showed a higher rate of cardiovascular risk factors and previous cardiovascular events (100% with cardiovascular risk factors and 50% with previous cardiovascular events). No patients with congenital or massive/torrential tricuspid valve regurgitation were observed.

38 patients (62%) died during follow-up. Overall, cardiovascular manifestations represented the most frequent causes of death (78.9% or 30/38), namely PH (39.4% or 15/38 patients; 8 in the SSc-PAH and 7 in SSc-PH groups, Table 1) and heart-related events (39.4% or 15/38 patients; 5 in SSc-PAH, 2 in group 2, and 8 in the SSc-PH group, Table 1). Cancer was the third cause of death (10.6% or 4/38 patients), 3 in SSc-PAH and 1 in the SSc-PH group, while deaths attributed to ILD were only 2 (5.3%) in the SSc-PH group. Finally, infections accounted for 2 deaths (5.3%), 1 in SSc-PAH and 1 in the SSc-PH group.

### Linear correlation

A significant correlation was found between reduced TAPSE/sPAP ratio and increased BNP (−0.388, *p* = 0.038), mPAP at RHC (−0.378, *p* = 0.003), PVR (−0.269, *p* = 0.036) and lower cardiac output (0.327, *p* = 0.025), 6MWD (0.274, *p* = 0.036), and DLCO (0.307, *p* = 0.017). To note, a correlation with late nailfolfd videocapillaroscopy (NVC) pattern (*p* < 0.001) was also found.

### SSc-PAH (group 1) vs. SSc- PH (group 3)

We compared SSc-PAH and SSc-PH patients (PH, Table 2).

**Table 2:** Comparison between SSc-PAH (group 1) and SSc-PH (group 3) patients.

Parameters	PAH (*n* = 29)	PH group 3 (*n* = 28)	*P*
Female (*n*,%)	89.7% (26/29)	78.6% (22/28)	0.297
Age (years)	68.7 ± 8,0	64.7 ± 11,1	0.122
Smokers (*n*,%)	34.5% (10/29)	53.6% (15/28)	0.186
Diabetes	6,9% (2/29)	7,1% (2/28)	1.000
Systemic hypertension	44,8% (13/29)	42,8% (12/28)	0.963
Overweight (BMI ≥25)	6,8% (2/29)	0% (0/61)	0.135
Dyslipidemia	34,4% (10/29)	35,7% (10/28)	1.000
Presence of ≥1 cardiovascular risk factor	55% (16/29)	53,5%(15/28)	0.878
Previous cardiovascular events	27,5% (8/29)	28% (8/28)	1.00
Disease duration (years)	12.5 ± 9.2	12.9 ± 7.2	0.849
Teleangectasia	82.8% (24/29)	64.2% (18/28)	0.141
Digital ulcers (*n*,%)	58.6%% (17/29)	53.6% (15/28)	0.793
Cutaneous subset			**0.021**
lcSSc	93.1% (27/29)	67.9% (19/28)	
dcSSc	6.9% (2/29)	32.1% (9/28)	
mRSS	7 ± 9	9 ± 5	0.453
ILD (*n*,%)	62.1% (18/29)	100% (28/28)	**0.001**
Limited ILD	62.1% (18/29)	0% (0/28)	**<0.001**
Significant ILD	0% (0/29)	100% (28/28)	**<0.001**
NYHA stage			0.061
I-II (*n*,%)	55.2% (16/29)	28.6% (8/28)	
III-IV (*n*,%)	44.8% (13/29)	71.4% (20/28)	
Autoimmunity			
ACA (*n*,%)	72.4% (21/29)	17.9% (5/28)	**<0.001**
ATA (*n*,%)	3.4% (1/29)	57.1% (16/28)	**<0.001**
ARA (*n*,%)	3.4% (1/29)	0% (0/28)	1.00
Electrocardiogram			
Right brunch block	48.3% (14/29)	46.4% (13/28)	1.00
Right axis deviation	6.9% (2/29)	17.9% (5/28)	0.253
Echocardiogram			
Left ventricle ejection fraction (%)	56 ± 5	56 ± 5	1.00
Diastolic disfunction (*n*,%)	57.1% (16/29)	55.6% (15/28)	1.00
Pericardial effusion (*n*,%)	13.8% (4/29)	14.3% (4/28)	1.00
TRV (m/s)	3.3 ± 0,6	3.3 ± 0,8	0.807
TAPSE (mm)	19.9 ± 3,6	20 ± 3,5	0.884
sPAP (mmHg)	53 ± 15	63 ± 27	0.110
TAPSE/sPAP ratio	0.40 ± 0.14	0.37 ± 0.17	0.526
PFTs			
FVC (%)	99 ± 19	67 ± 19	**<0.001**
DLCO (%)	43 ± 16	28 ± 12	**<0.001**
FVC/DLCO ratio	2.5 ± 1,0	1.7 ± 0,9	0.767
Desaturation at 6MWT	31% (9/29)	60.7% (17/28)	**0.034**
6MWD (mt)	392 ± 118	389 ± 132	0.935
Videocapillaroscopy			
Aspecific (*n*,%)	0% (0/29)	0% (0/28)	
Early (*n*,%)	20.7% (6/29)	36% (1/28)	0.102
Active (*n*,%)	41.7% (12/29)	32.1% (9/28)	0.585
Late (*n*,%)	37.9% (11/29)	64.3% (18/28)	0.065
Blood chemistry analysis			
BNP (pg/ml)	202 ± 178	471 ± 470	0.065
Serum urate elevation (*n*,%)	44.8% (13/29)	42.9% (12/28)	1.00
RHC			
mPAP (mmHg)	34 ± 10	35 ± 13	0.739
PVR	5.9 ± 4,1	5.8 ± 3.1	0.893
PAWP	11.1 ± 4.5	13.6 ± 7.4	0.119
Cardiac index	2.6 ± 0.7	2.6 ± 0.7	0.890
Cardiac output	4.5 ± 1.6	4.2 ± 1.1	0.538
Outcomes			
Death	58.6% (17/29)	67.9% (18/28)	0.585
Survival (months)	55.3 ± 31.2	25 ± 19	**0.050**
Cardiovascular events	64% (18/29)	67% (19/28)	1.00
Heart failure	83% (15/18)	94% (18/19)	
Arrytmias	28% (5/18)	21% (4/19)	
Stroke/Transient ischemic events	0% (0/18)	10,5% (2/19)	
Pulmonary thomboembolism	5,5% (1/18)	10,5% (2/19)	
Sudden death	11% (2/18)	16% (3/19)	
Therapies			
Iloprost i.v.	75,9% (22/29)	71,4% (20/28)	0,770
Iloprost inhaled	0% (0/29)	7% (4/24)	0,052
CCbs (*n*,%)	41,4% (12/29)	60,7% (17/28)	0,189
B-Blockers (*n*,%)	27,6% (8/29)	7,1% (2/28)	0,079
ERA(*n*,%)	82,8% (24/29)	64,3% (18/28)	0,141
Bosentan (*n*,%)	55,2% (16/29)	39,3% (11/28)	0,292
Macitentan (*n*,%)	41,4% (12/29)	39,3% (11/28)	1,00
PDEi (*n*,%)	51,7% (15/29)	53,6% (15/28)	1,00
Selexipag (*n*,%)	10,3% (3/29)	3,6% (1/28)	0,611
Death cause			
Heart	29.4% (5/17)	42.1% (8/19)	
PH	47% (8/17)	36.8% (7/19)	
ILD	0% (0/17)	10.5% (2/19)	
Infection	5.9% (1/17)	5.3% (1/19)	
Cancer	17.7% (3/17)	5.3% (1/19)	

PH, pulmonary hypertension; ILD, interstitial lung disease; mRSS, modified Rodnan Skin Score; NYHA, New York Heart Association; ANA, anti-nucleus antibodies; ACA, anti.centromere antibodies, ATA, anti-topoisomerase I antibodies; ARA, anti-RNA polymerase III antibodies; RV, right ventricle; TRV, tricuspid regurgitant velocity, TAPSE, tricuspid annular plane systolic excursion; sPAP, systolic pulmonary arterial pressure; FVC, force vital capacity; DLCO, diffusing capacity of the lung for carbon monoxide; 6MWT, six minutes walking test, 6MWD, six minutes walking distance; BNP, brain natriuretic peptide; CCBs, calcium channel blockers; ERA, endothelin receptor antagonists; PDEi, phosphodiesterase inhibitors; mPAP, mean pulmonary arterial pressure; PVR, pulmonary vascular resistance; PAWP, pulmonary arterial wedge pressure.

Bold values were statistically significant.

In both groups, lcSSc was the most common cutaneous subset (67.9% or 19/28 patients), yet a higher frequency of dcSSc (32.1% vs. 6.9%, *p* = 0.021) and ATA (57.1% vs. 3.4%, *p* = <0.001) were found in SSc-PH compared to the SSc-PAH group. The mRSS was slightly higher in the SSc-PH group, yet with no significant difference (9 ± 5 SD vs. 7 ± 9 SD, *p* = 0.453). Late SSc was the most common NVC pattern in SSc-PH (early pattern 3.6%, active pattern 32.1%, late pattern 64.3%), while in SSc-PAH it was the active pattern (early pattern 20.7%, active pattern 41.4%, late pattern 37.9%).

No significant differences regarding the presence of cardiovascular risk factors (namely smoking, being overweight, diabetes, systemic hypertension and dyslipidemia) and cardiovascular events occurring before PH diagnosis, were seen among these two groups (SSc-PAH 27.5% vs. SSc-PH 28%, *p* = 1.000).

SSc-PH patients had a worse NYHA class (statistical trend, *p* = 0.061) and PFTs, with lower TLC (67% ± 19 SD vs. 99% ± 19 SD, *p* < 0.001), FVC (75% ± 16SD vs. 107% ± 15 SD, *p* < 0.001) and DLCO (28% ± 12 SD vs. 43% ± 16 SD, *p* < 0.001) compared to SSc-PAH; moreover, higher rates of desaturation at 6MWT (31.0% vs. 60.7%, *p* = 0.034) were seen in this group.

BNP levels were tendentially higher in SSc-PH, yet without statistical significance (471 pg/ml ± 470 SD vs. 202 pg/ml ± 178 SD, *p* = 0.065).

Regarding echocardiographic and hemodynamic parameters, no significant differences were observed.

No significant differences were found in death and the rates of cardiovascular events, while SSc-PH patients had a significant lower survival interval time (25 months ± 19 SD vs. 55.3 months ±31.2 SD, *p* = 0.05). In PAH patients, the main cause of death was PH itself (47% or 8/15 patients), in SSc-PH group, while death was mainly attributed to heart related events (42.1% or 8/19 patients). Notably, death related to ILD was recorded only in 2 patients (10.5%).

No differences were found in the therapies administrated during follow-up, with the exception of inhaled Iloprost in 4 SSc-PH patients (7%), compared to none from the PAH group.

## Survival

### Univariate and multivariate Cox analysis

In the Univariate Cox regression analysis, the TAPSE/sPAP ratio <0.32 mm/mmHg, male gender, significant ILD, PVR>5 WU and NYHA functional class III-IV, were substantially associated with mortality in overall PH population (Table 3).

**Table 3:** Univariable and multivariable Cox regression analyses for independent predictors of mortality.

Univariate analysis				Multivariate analysis		
Parameters	HR	IC 95%	*p*	HR	IC 95%	*p*
Male sex	2.682	1.195–6.019	**0**.**017**	4.095	1.614–10.386	**0**.**003**
Age	0.988	0.958–1.020	0.462			
Disease duration	0.990	0.952–1.029	0.610			
Digital ulcers	1.052	0.542–2.042	0.881			
mRSS	0.989	0.942–1.038	0.658			
ATA	1.222	0.588–2.537	0.591			
Late NVC pattern	1.607	0.836–3.089	0.55			
Significant ILD	2.483	1.161–5.310	**0**.**019**	2.407	1.082–5.354	**0**.**031**
NYHA class III-IV	2.261	1.261–5.446	**0**.**010**	0.735	0.232–2.335	0.602
Pericardial effusion	1.821	0.849–3.908	0.124			
TAPSE/sPAP <0.32	2.365	1.230–4.549	**0**.**010**	2.439	1.143–5.205	**0**.**021**
PVR >5	2.248	1.115–4.533	**0**.**024**	1.232	0.500–3.036	0.651
DLCO	0.980	0.958–1.002	0.076			
Cardiac Index	0.677	0.166–1.176	0.166			
BNP	1.001	1.000–1.003	0.076			
Serum urate elevation	1.413	0.743–2.688	0.292			

Bold values were statistically significant.

In the multivariate analysis, the TAPSE/sPAP ratio <0.32 mm/mmHg outlined a significant independent association with mortality [HR 2,439 (95% CI 1,143–5,205); *p* = 0.021].

Male gender [HR 4.095 (95% CI 1.614–10.386); *p* = 0.003] and the presence of significant ILD [HR 2.407 (95% CI 1.082–5.354); *p* = 0.031] were also identified as independent predictors of mortality, while the correlation between PVR>5 WU, NYHA class III-IV and mortality was not confirmed (Table 3).

### Kaplan Meier curves

**Figure 2:**
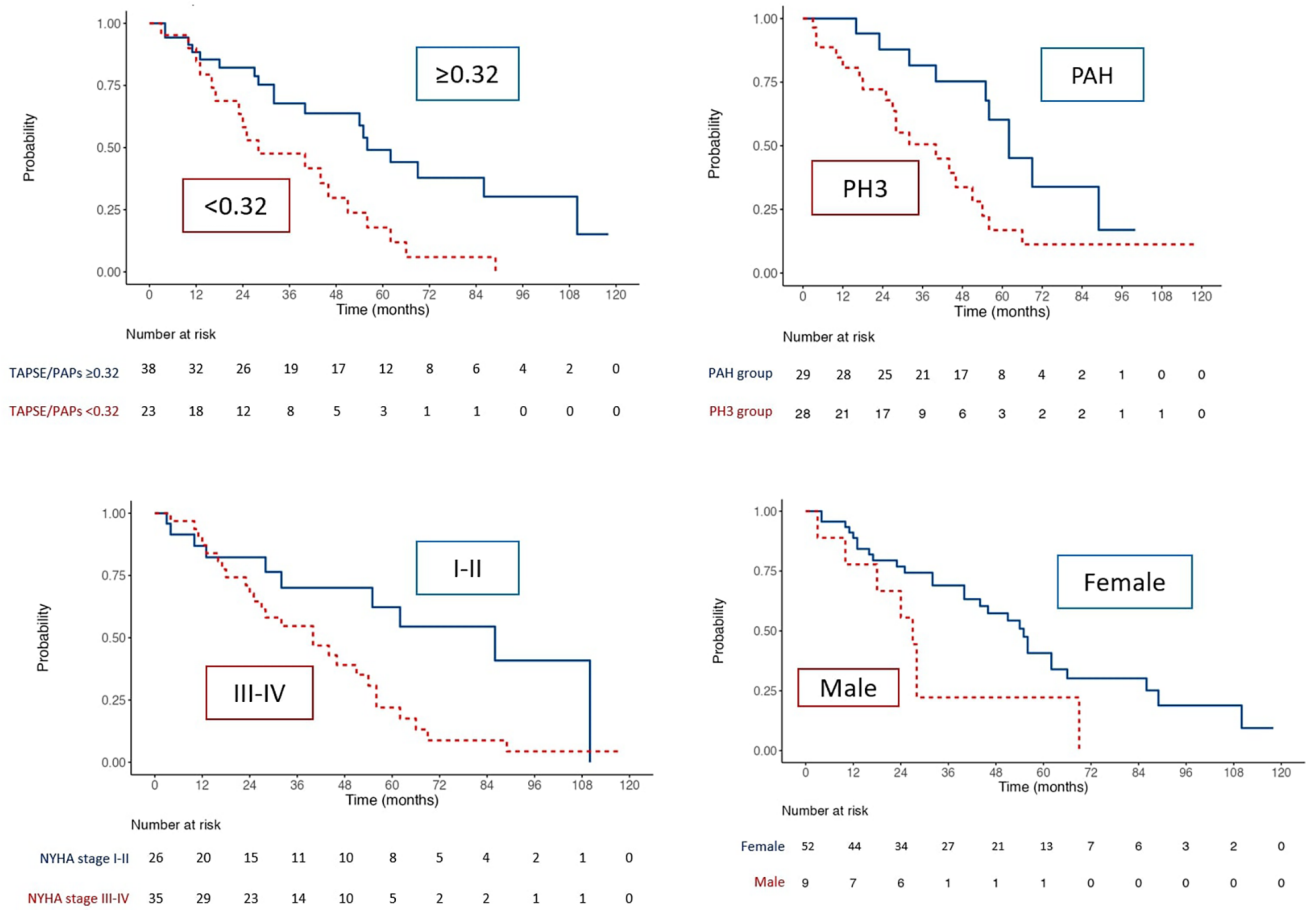
Kaplan–Meier analysis for survival for TAPSE/sPAP ratio ≤0.32, PH group, NYHA class and sex.

Kaplan Meier curves of the whole population (Figure 2) outlined a reduced survival in SSc patients with TAPSE/sPAP ratio ≤0.32 mm/mmHg (Log Rank χ2 8.068, *p* = 0.005, Figure 2) and in patients of the SSc-PH group (Log Rank χ2 5.792, *p* = 0.016), NYHA class III-IV (Log Rank χ2 5.792, *p* = 0.016) and male gender (Log Rank χ2 5.986, *p* = 0.014). In addition, the presence of PVR >5WU (Log Rank χ2 7.412, *p* = 0.006), was associated with worse survival.

## Cardiovascular events

### Univariate and multivariate Cox analysis

In the Univariate Cox regression analysis, the TAPSE/sPAP ratio <0.32 mm/mmHg, male gender, significant ILD, BNP and NYHA functional class III-IV were substantially associated with the presence of cardiovascular events (Table 4).

**Table 4:** Univariable and multivariable Cox regression analyses for independent predictors of cardiovascular events.

Univariate analysis				Multivariate analysis		
Parameters	HR	IC 95%	*P*	HR	IC 95%	*p*
Male sex	3.411	1.554–7.488	**0** **.** **002**	3.878	1.34–9.804	**0**.**004**
Age	0.988	0.959–1.024	0.606			
Disease duration	0.990	0.949–1.029	0.561			
Digital ulcers	1.302	0.651–2.604	0.455			
mRSS	1.014	0.973–1.057	0.506			
ATA	1.406	0.687–2.876	0.351			
Late NVC pattern	1.526	0.788–2.953	0.210			
Significant ILD	2.465	1.150–5.282	**0**.**020**	2.500	1.124–5.564	**0**.**025**
NYHA class III-IV	2.050	1.118–4.129	**0**.**044**	0.603	0.217–1.674	0.401
Pericardial effusion	2.007	0.927–4.345	0.077			
TAPSE/sPAP <0.32	2.127	1.091–4.145	**0**.**027**	2.439	1.803–4.943	**0**.**030**
PVR >5	1.633	0.818 −3.259	0.164			
DLCO	0.980	0.958–1.003	0.082			
Cardiac index	0.752	0.432–1.307	0.312			
BNP	1.002	1.002–1.003	**0**.**020**			
Serum urate elevation	1.318	0.688–2.527	0.405			

Bold values were statistically significant.

The multivariate analysis confirmed the TAPSE/sPAP ratio <0.32 mm/mmHg [HR 2.439 (95% CI 1.803–4.943); *p* = 0.030], male gender [HR 3.878 (95% CI 1.34–9.804); *p* = 0.004] and the presence of significant ILD [HR 2.500 (95% CI 1.124–5.564); *p* = 0.025] as independent predictors of cardiovascular events (Table 4).

### Kaplan Meier curves

**Figure 3:**
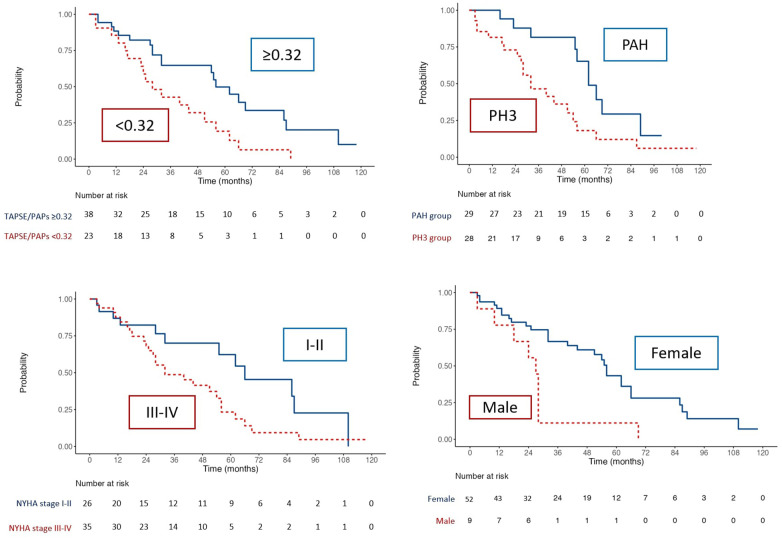
Kaplan–Meier analysis for free- cardiovascular events survival for TAPSE/sPAP ratio ≤0.32, PH group, NYHA class and sex.

Kaplan Meir curves of the overall population of patients (Figure 3) highlighted that TAPSE/sPAP ≤0.32 mm/mmHg (Log Rank χ2 5.207, *p* = 0.022), SSc-PH (Log Rank χ2 5.807, *p* = 0.016), NYHA functional class III-IV (Log Rank χ2 4.258, *p* = 0.039) and male (Log Rank χ2 10.664, *p* = 0.001), were associated with reduced free-cardiovascular events survival.

## Discussion

Our study is in keeping with the few studies on the potential role of TAPSE/PAPs ratio in predicting worse outcomes in SSc-PH patients (22–25). Indeed, abnormally low values of said parameter were inversely correlated with increased mPAP at RHC, serum BNP, number of cardiovascular manifestations, and prevalence of late NVC pattern that characterise this disease setting.

The detection of TAPSE/PAPs ratio <0.32 mm/mmHg was an independent predictor of mortality and cardiovascular events in SSc-PH patients.

Regarding the other clinical, serological, hemodynamic and instrumental parameters, including male gender, the presence significant ILD, worse NYHA functional class and severe impairment of PVR (>5WU), were associated with mortality and cardiovascular events, however, the former two were confirmed during multivariate analysis.

The comparison between SSc-PAH and SSc-PH subgroups revealed a significantly higher incidence of lcSSC, ACA positive, and the presence of capillaroscopic active pattern in the first subgroup, while SSc-PH correlated with dcSSc, ATA positive individuals, and the presence of late NVC pattern. To note, SSc-PH showed a significantly higher mortality rate compared to the subset of SSc-PAH patients and—more frequently—secondary to different heart related events (HF, sudden death, arrhythmias, etc.), while in deceased individuals with SSc-PAH, the main cause of death was PH.

At the end of follow-up, almost two thirds of SSc patients died (62% or 38/61 patients), PH with/without other cardiovascular events was responsible for death in more than 3/4 individuals (79% or 30/38 patients). Interestingly, cancer was the third reason of death, while ILD was seldomly considered as the main cause of death.

There is growing evidence regarding the importance of the TAPSE/PAPs ratio in predicting the prognosis of heart failure patients, whether secondary to PH or not. Recently, larger study have confirmed that an impaired RV-pulmonary artery coupling, assessed through TAPSE/sPAP ratio, in associated with lower survival and higher risk of hospitalization in patients with PH associated with pulmonary embolism, heart failure with preserved ejection fraction and also in heart transplant patients (26–28).

Nonetheless, some considerations should be done, in particular, because the normal limits and physiologic correlates of TAPSE are not well known, even in healthy subjects. Some studies reports that TAPSE, PAPS and TAPSE/PAPAS demonstrates an association with advancing age and impaired diastolic function, even if the relationship between TAPSE and PAPs is relatively well preserved across the age groups (29–31).

However, the literature on SSc is still limited. Existing studies are few and often characterised by small heterogeneous sample sizes, as well as by limited number of cardiopulmonary clinical parameters. Despite being retrospective, our work aims to analyse the disease in its complexity, also considering the possible contribution of ILD and scleroderma cardiomyopathy to TAPSE/PAPs ratio alterations.

The prognostic significance of TAPSE/PAPs ratio in SSc, alone and combined with NTproANP (Atrial Natriuretic Peptide), has recently been evaluated in a few studies (23–32). Nevertheless, the TAPSE/sPAP ratio was included in the three-strata model risk assessment of mortality at 1 year for PAH in the 2022 ESC/ERS guidelines (16).

A prospective study by Lai et al. (24), enrolling 61 SSc patients with PAH, demonstrated that TAPSE/sPAP <0.19 mm/mmHg appeared to be a reliable indicator in the stratification of patients with high risk of all-causes death and clinical worsening. In our work, TAPSE/PAPs<0.32 mm/mmHg was significantly and independently associated with all the causes of mortality and cardiovascular events. However, we were unable to corroborate the cut-off of <0.19 mm/mmHg, due to a lack of patients with TAPSE/PAPs ratio under this threshold.

Our findings are also consistent with the recent study by Grimaldi et al. (23), who retrospectively assessed 70 SSc patients with no signs or symptoms of PH. A reduced TAPSE/sPAP ratio, alone and combined with elevated NT-pro-ANP, revealed to be independently associated with cardiovascular events and deaths during a 6-year follow-up. Interestingly, another study highlighted that ANP could predict disease progression and the development of digital ulcers in systemic sclerosis patients (34).

Finally, similar results were obtained from larger studies. In particular, a recent retrospective study involving 2,555 SSc patients from the European Scleroderma Trials & Research Group (EUSTAR) cohort, confirmed the predictive role of TAPSE/PAPs ratio <0.55 mm/mmHg for PAH diagnosis and for all-cause mortality in SSc patients (25).

Our study included all SSc patients, both incident and prevalent, who underwent RHC from October 2013 to October 2023. PH typically occurs as a later manifestation of the disease in SSc. On average, PH is diagnosed approximately from 5 to 10 years after the onset of symptoms of systemic sclerosis, however this can vary widely among patients (12). In our population the average disease duration at diagnosis of PH was 12.9 years, and in 80% of the patients (49/61) PH was diagnosed after the fifth year of disease. In one case, PH was the onset symptom of SSc.

Despite the development of a screening algorithm for early diagnosis of PH and the introduction of several new drugs, the mortality rate of both SSc-PAH and SSc-PH has not reduced in the modern era when compared to idiopathic PAH (34). Indeed, in our cohort, PH and other cardiovascular events were the main causes of death. We can hypothesise that SSc with either PAH or PH may represent a particular subgroup of patients in which abnormally increased pulmonary pressure *per se* may be the main adverse prognostic factor. A more or less prominent role of lung fibrosis in SSc-PH may explain the worse outcome observed in this subgroup compared to SSc-PAH, as well as in the whole series of patients with SSc complicated by PH and to patients with idiopathic PAH (7). It has also been suggested that the poor prognosis of both SSc-PAH and SSc-PH could mainly be due to a more prominent maladaptive remodelling of the RV and concomitant scleroderma cardiomyopathy (35). SSc patients have intrinsic myocardial dysfunction and impaired pulmonary macro- and microvascular anatomy and function, which increases when developing pulmonary hypertension. RV-PA uncoupling in SSc patients with PAH and PH correlates with remodelling of the right ventricle and pulmonary artery vessels. Therefore, it is crucial to investigate SSc-RV function and RV-PA coupling in order to explain the differences in the outcome between SSc-PH and idiopathic PAH and to identify SSc patients at high risk of mortality. The TAPSE/sPAP ratio assesses ventriculoarterial coupling and refers to the cardiopulmonary unit. In fact, it represents the relationship between preload (TAPSE) and afterload (sPAP).

RV dysfunction in SSc may be in part independent of PAH (36, 37), and it can be linked to irreversible fibrotic changes, secondary to recurrent ischemic-reperfusion cycles which are responsible for typical scleroderma cardiomyopathy (38, 39). Several studies emphasised that SSc-PH patients show a more severe peripheral microcirculatory injury, characterised by a progressive loss of capillaries and complete vascular remodelling, corresponding to the *late scleroderma pattern* (40, 41). A predictive role of NVC pattern for future cardiovascular events and mortality has been proposed (42, 43). To our knowledge, this is the first study in which a significant correlation between a lower TAPSE/PAPs ratio and *late scleroderma pattern* at NVC was found. We also observed a significantly higher rate of patients with late NVC pattern in the deceased group compared to survived patients, albeit NVC pattern was not identified as an independent predictor of mortality.

Increased mortality was also seen in male patients and patients with PVR >5 WU, NYHA class III-IV and presence of significant ILD. Male gender is a well-known risk factor in SSc, and it has mainly been associated with the diffuse cutaneous subset, progressive ILD and poor survival rates (38). A recent metanalysis by Barkhane et al. (46) confirmed an increased risk of mortality in SSc patients with higher PVR and worse NYHA functional class, supporting the importance of assessing and managing PVR and cardiac index in patients with SSc-PH. Our study also found a correlation between reduced TAPSE/PAPs and increased PVR.

It should be noted that the available studies only focus on the role of TAPSE/sPAP ratio in patients with PAH, while patients with significant ILD were excluded during enrolment. To our knowledge, this is the first study in which TAPSE/sPAP ratio was assessed in SSc patients with significant ILD and a comparison between the PAH and PH groups was made.

In our cohort no significant differences were observed between SSc-PAH and SSc-PH groups regarding the presence of cardiovascular risk factors and cardiovascular events prior to the diagnosis of PH. As expected, only the 4 patients with PH of group 2 showed a higher rate of both cardiovascular risk factors (100%) and previous cardiovascular events (50%). Moreover, no SS-PAH nor SSC-PH patients had moderate-severe valvular diseases, while 50% (2/4) of PH patients from group 2 had moderate-severe mitral regurgitation. This was expected, since PH of group 2 is related to left heart diseases, which are frequently influenced by the presence of cardiovascular risk factors, previous cardiovascular events and left valvular diseases (44).

The presence of PH in patients with significant lung parenchymal impairment may be underestimated since RHC is rarely performed on these patients. However, the development of PH in the context of lung disease significantly affects the quality of life and survival of the patients, as recently observed in COVID-19 survivors (47, 48).

Furthermore, SSc-ILD-PH patients suffer from multifactorial factors, namely interstitial pulmonary complications, macroangiopathic and microangiopathic cardiomyopathy, modification and remodelling of the pulmonary vascular circulation. Indeed, in our cohort, SSc-PH patients showed a lower survival rate compared to the SSc-PAH group and the presence of significant ILD was identified as an independent predictor of mortality and cardiovascular events. Our results are concordant with data from Spanish and German registries, which revealed that SSc-ILD-PH patients are characterised by lower survival compared to either SSc-ILD and SSc-PAH patients, as well by worse hemodynamic profile (namely increased PVR and reduced CI) (49, 50).

Moreover, in our cohort serum BNP levels were tendentially higher (471 ± 470 pg/ml vs. 202 ± 178 pg/ml, *p* = 0.065) in SSc-PH patients, and—to note—heart-related events and PH were the main causes of death in this group. These findings may be indicative of RV-PA uncoupling, resulting in RV maladaptation. SSc-PH patients of our cohort also showed severely increased PVR and reduced TAPSE/PAPs ratio, which may represent a proper surrogate of RV-PA coupling impairment.

Notably, in our population no differences between PAH-specific therapies—administered during follow-up—and between PAH and PH patients were found, with 64.3% (18/28) taking ERAs and 53.6% (15/28) taking PDEi, despite the 2022 ESC/ERS guidelines stating that PAH-target therapies should only be reserved for patients with PAH (group 1). The RESCLE registry had comparable data, showing that up-front combination therapy was administered in 59.8% and 61.7% of patients with and without ILD, respectively, raising the issue regarding the extensive off-label use of vasodilatator therapies in SSc-PH-ILD patients and the increasing evidence that early treatment may improve outcomes (49). Therefore the administration of PAH specific therapies in SSc-ILD-PH patients, also taking into account the difficulty in the assignment of a specific PH group, must be considered as an unmet need and therefore perspective randomised controlled trials are needed to clarify the issue.

Overall, the TAPSE/PAPs ratio, along with other clinical and prognostic parameters, highlighted a worse outcome of SSc-PH compared to the SSc-PAH patients’ subgroup, which is likely due to a more severe disease variant as a whole. Indeed, the SSc-PH patients showed a higher prevalence of dcSSc, concomitant lung and heart involvement, serum ATA, late capillaroscopic pattern, together with a significantly lower survival rate.

### Limitation

The present study has some limitations. The first concerns the study design that is a retrospective cohort analysis. The second regards the low number of patients enrolled; however, they are well characterized from both a rheumatological and cardiological point of view. Furthermore, this is a monocentric study but all the patients come from a scleroderma unit which is a point of reference in the Italian panorama, all the cardiological evaluations are carried out by cardiologists specialized in cardiac involvement secondary to connective tissue diseases and having centralized the cardiological evaluation guarantees the homogeneity of the assessment. Moreover, the numerosity of the sample and the disease characteristics, in line with other cohorts, support the representativeness of our population. The absence of interobserver agreement for echocardiographic measurements could represents another limit of our analysis. However, the parameters used are based on standardized, reliable protocols. Moreover, tricuspid annular plane systolic excursion (S’) and RVOT acceleration time, despite being usually assessed, were not reported due to their unavailability in most records; as well detailed assessments of left ventricular diastolic function and left atrial volume for evaluating scleroderma cardiac complications.

## Conclusions

Despite the limitations of our retrospective cohort study, the observed findings underlined the predictive role of reduced TAPSE/PAPs ratio, as well of male gender and presence of significant ILD for both cardiovascular events and mortality in SSc patients complicated by pulmonary hypertension. In-deep investigations might clarify the actual relationship between the severity of microangiopathic cardiomyopathy and altered RV-PA coupling in the SSc complicated by PH, with/without significant lung fibrosis.

## Data Availability

The original contributions presented in the study are included in the article/Supplementary Material, further inquiries can be directed to the corresponding author.
